# Case Report: Remarkable breakthrough: successful treatment of a rare intracranial mesenchymal, FET::CREB fusion-positive tumor treated with patient-tailored multimodal therapy

**DOI:** 10.3389/fonc.2023.1203994

**Published:** 2023-11-29

**Authors:** Federica D’Antonio, Sabrina Rossi, Isabella Giovannoni, Rita Alaggio, Andrea Carai, Giuseppe M. Milano, Antonella Cacchione, Alessandra Cancellieri, Marco Gessi, Manila Antonelli, Giovanna S. Colafati, Giacomina Megaro, Sabina Vennarini, Angela Mastronuzzi

**Affiliations:** ^1^ Department of Experimental Medicine, Faculty of Medicine and Dentistry, Sapienza University of Rome, Rome, Italy; ^2^ Department of Pathology, Bambino Gesù Children’s Hospital (IRCCS), Rome, Italy; ^3^ Unit of Neurorehabilitation, Department of Intensive Neurorehabilitation and Robotics, Bambino Gesù Children’s Hospital (IRCCS), Rome, Italy; ^4^ Department of Oncohematology, Hematopoietic Transplantation, and Cell Therapy, Bambino Gesù Children’s Hospital (IRCCS), Rome, Italy; ^5^ Institute of General Pathology, Agostino Gemelli University Polyclinic (IRCCS), Rome, Italy; ^6^ Department of Radiological, Oncological, and Pathological Anatomy Sciences, Faculty of Medicine and Dentistry, Sapienza University of Rome, Rome, Italy; ^7^ Department of Diagnostic Imaging, Bambino Gesù Children’s Hospital (IRCCS), Rome, Italy; ^8^ Radiotherapy Department, Fondazione IRCCS Istituto Nazionale Dei Tumori, Milan, Italy

**Keywords:** intracranial mesenchymal tumor, FET::CREB gene fusion, molecular analysis, rare cancers, challenging diagnosis, immunotherapy, multimodal tailored therapy

## Abstract

**Background:**

Intracranial mesenchymal tumors are a rare type of neoplasm (0.3% of all soft tissue tumors) characterized by a fusion of a *FET* family gene (usually *EWSR1*, rarely *FUS*) to *CREB* family genes (*CREB1, ATF1*, and *CREM*) with a slow-growing and favorable prognosis. Mesenchymal tumors are most frequently localized in the subcutaneous tissue (typically in the limbs and hands) of young adults and have rarely been diagnosed in the central nervous system. Surgery is the gold standard treatment; adjuvant radiation therapy and chemotherapy with sarcoma-based regimens have been used in rare cases when complete surgical excision was not recommended. In terms of prognosis, these tumors show a tendency for local relapse. The longest patient outcomes reported in the literature are five years.

**Case description:**

This case describes a 27-year-old woman with unconventional extracranial metastatic sites of myxoid intracranial mesenchymal tumor *FET::CREB* fusion-positive and high expression of PD-1 (40%) and PD-L1 (30%). Based on clinical, molecular, and histological characteristics, she underwent various local and systemic therapies, including surgery, proton beam therapy, the use of immune checkpoint inhibitors, and chemotherapy. These treatments led to a complete remission of the disease after eight years from tumor diagnosis.

**Conclusions:**

Our case sheds light on the importance of precision medicine and tailored therapy to explore new treatment opportunities for rare or unknown tumor entities.

## 1 Introduction

Mesenchymal tumors are rare soft connective tissue neoplasms (1, 2). These tumors may arise in all organs originating from mesodermal precursor cells and also in the central nervous system (CNS), specifically from the meninges and rarely from CNS parenchyma (2, 3).

The *FET* family (usually *EWSR1*, less frequently *FUS*) gene’s rearrangements with *CREB* family genes (*CREB1*, *ATF1*, and *CREM*) have been identified as characterizing a specific group of mesenchymal tumors called angiomatoid fibrous histiocytomas (AFH) (4). These are uncommon soft tissue tumors (typically found in the limbs, hands feet, or pelvis of children and young adults), with an incidence of < 0.5% and are classified as low-intermediate growing neoplasms with a favorable prognosis (2, 5).

More recently, intracranial AFH has been described as an intracranial mesenchymal tumor (6–8). AFH is usually treated with surgical removal, and only in cases of incomplete resection may adjuvant radiation therapy or chemotherapy be necessary (5). We hereby present a unique case of an intracranial mesenchymal tumor with *EWSR1::CREM* fusion transcript with extra CNS metastatic spreads, treated with different local and systemic therapies, leading to prolonged complete remission of the disease after two years of treatment discontinuation and after eight years from tumor diagnosis.

## 2 Case report

**Figure 1:**
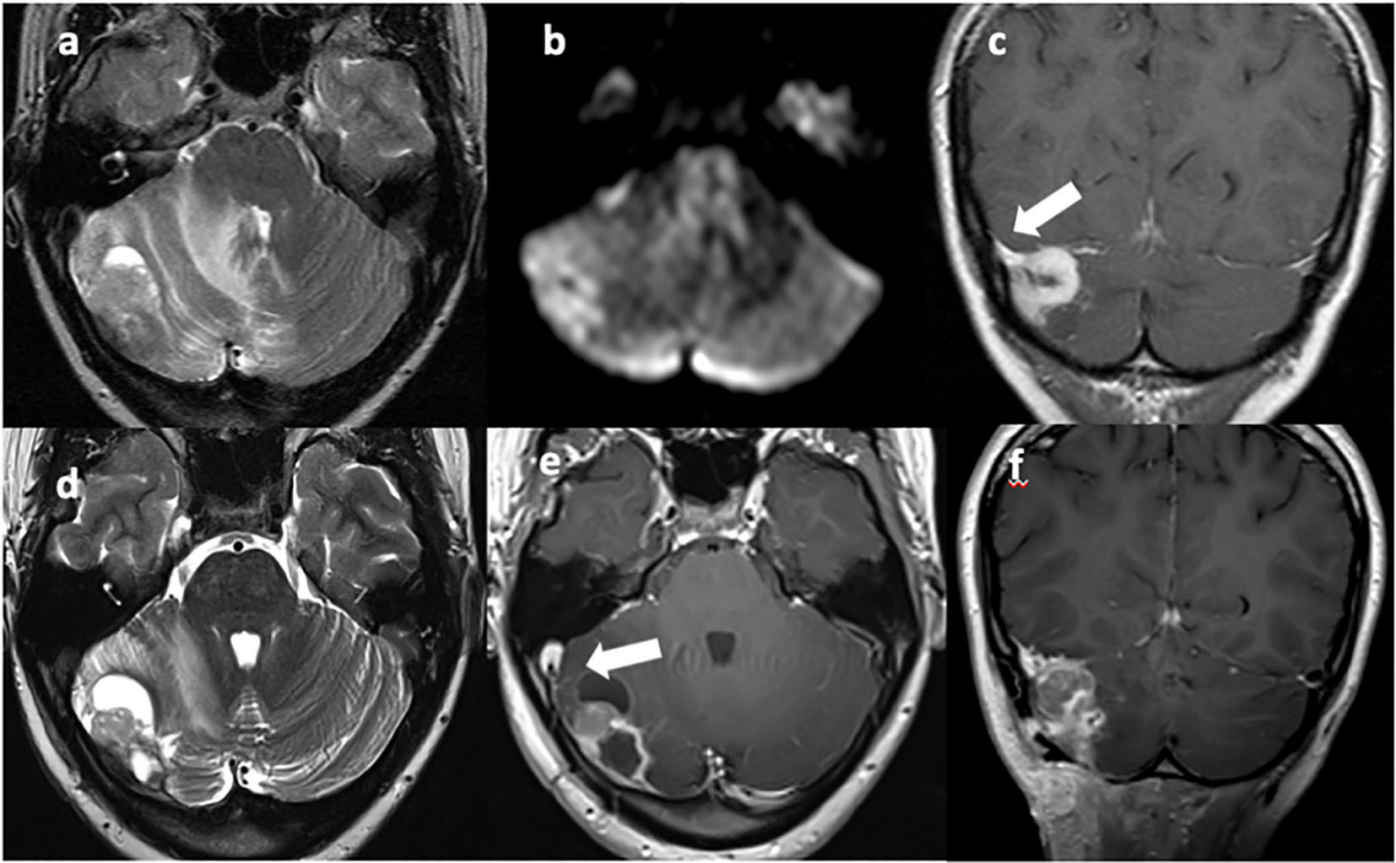
T2w axial **(A, D)** and T1w axial **(E)** and coronal **(C, F)** MRI images show a vascularized solid-cystic edematous lesion in the right cerebellar hemisphere, in continuity with the right transverse sinus [**(C)**, arrow]. Diffusion restriction is present due to the high cellularity of the neoplastic tissue **(B)**. Sinus thrombosis is also present [**(E)**, arrow].

**Figure 2:**
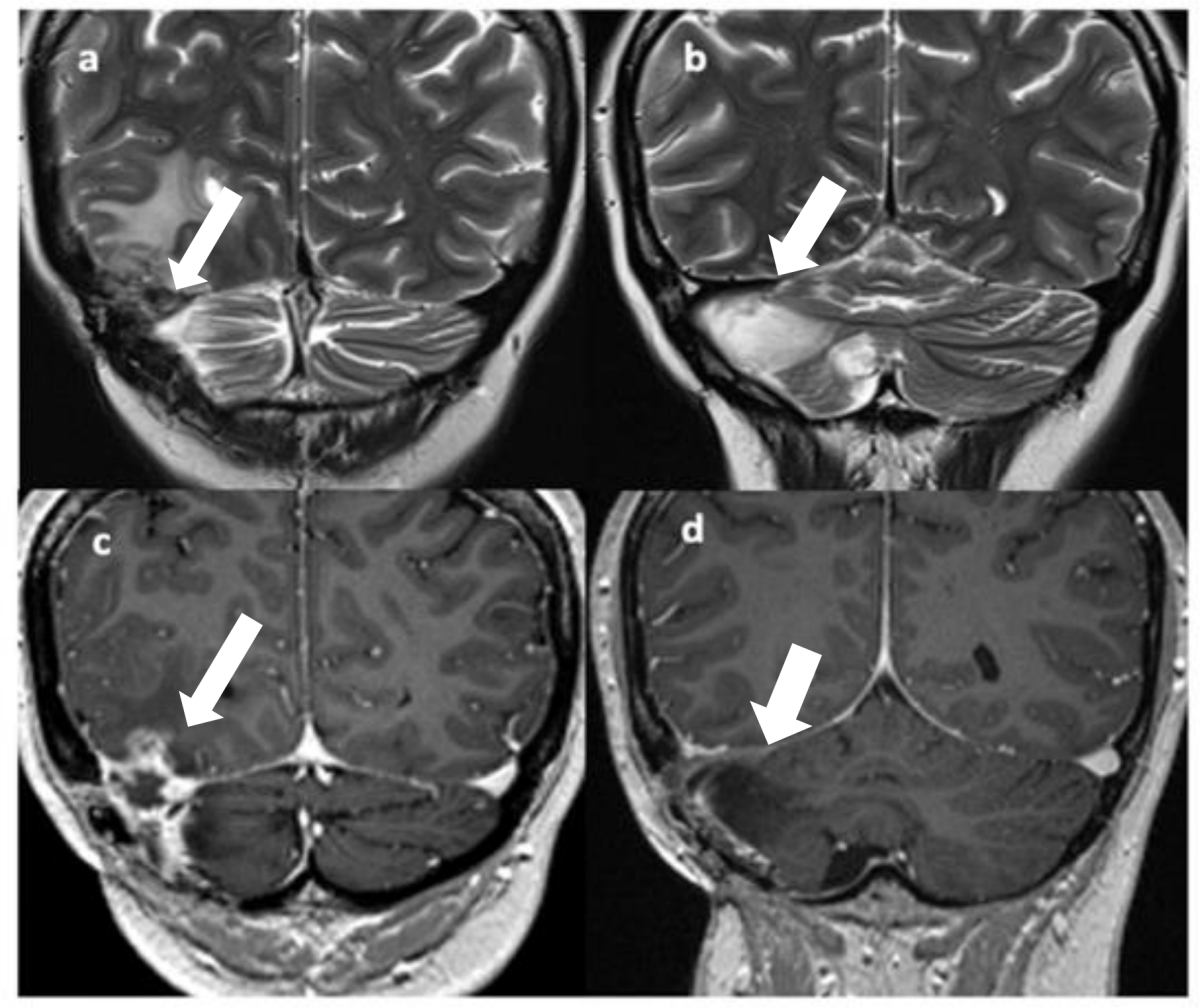
T2 **(A, B)** and Gd T1 TSE **(C, D)** coronal images show disease recurrence with a solid-cystic, edematous lesion in the right temporo-occipital and right cerebellar regions with involvement of the right transverse sinus [**(A, C)**, arrows]; gliotic-malacic findings coexist in the right hemicerebellar region [**(B, D)**, arrows].

**Figure 3:**
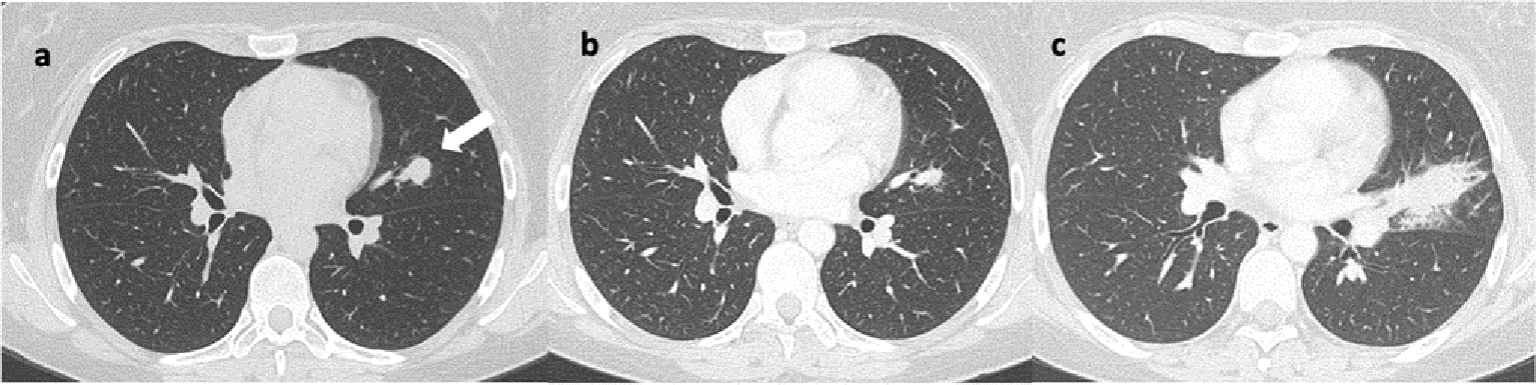
Axial lung CT scans reveal: **(A)** a nodular lesion in the upper lingular segment, indicated by the arrow. Image **(B)** shows a reduction in size at the 14-month follow-up after initiation of immunotherapy. Image **(C)** shows a significant increase in size at the 6-month follow-up after discontinuation of immunotherapy, characterized by spiculated margins.

**Figure 4:**
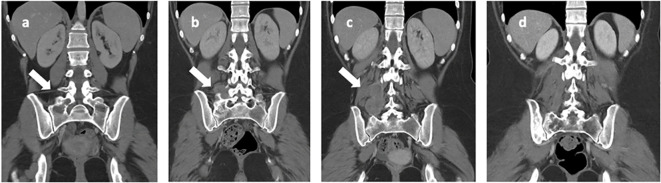
Iliac bone CT scans highlight: **(A)** a lesion with lytic features located in the right iliac wing and sacral wing (indicated by the arrow) at the onset of bone metastasis. In images **(B, C)**, there is a progressive increase in the extension observed in the right paravertebral soft tissue (indicated by the arrows), as seen at the time of discontinuation of nivolumab. Image **(D)** depicts the nearly complete resolution observed at the last follow-up, which occurred 8 years after disease onset.

**Figure 5:**
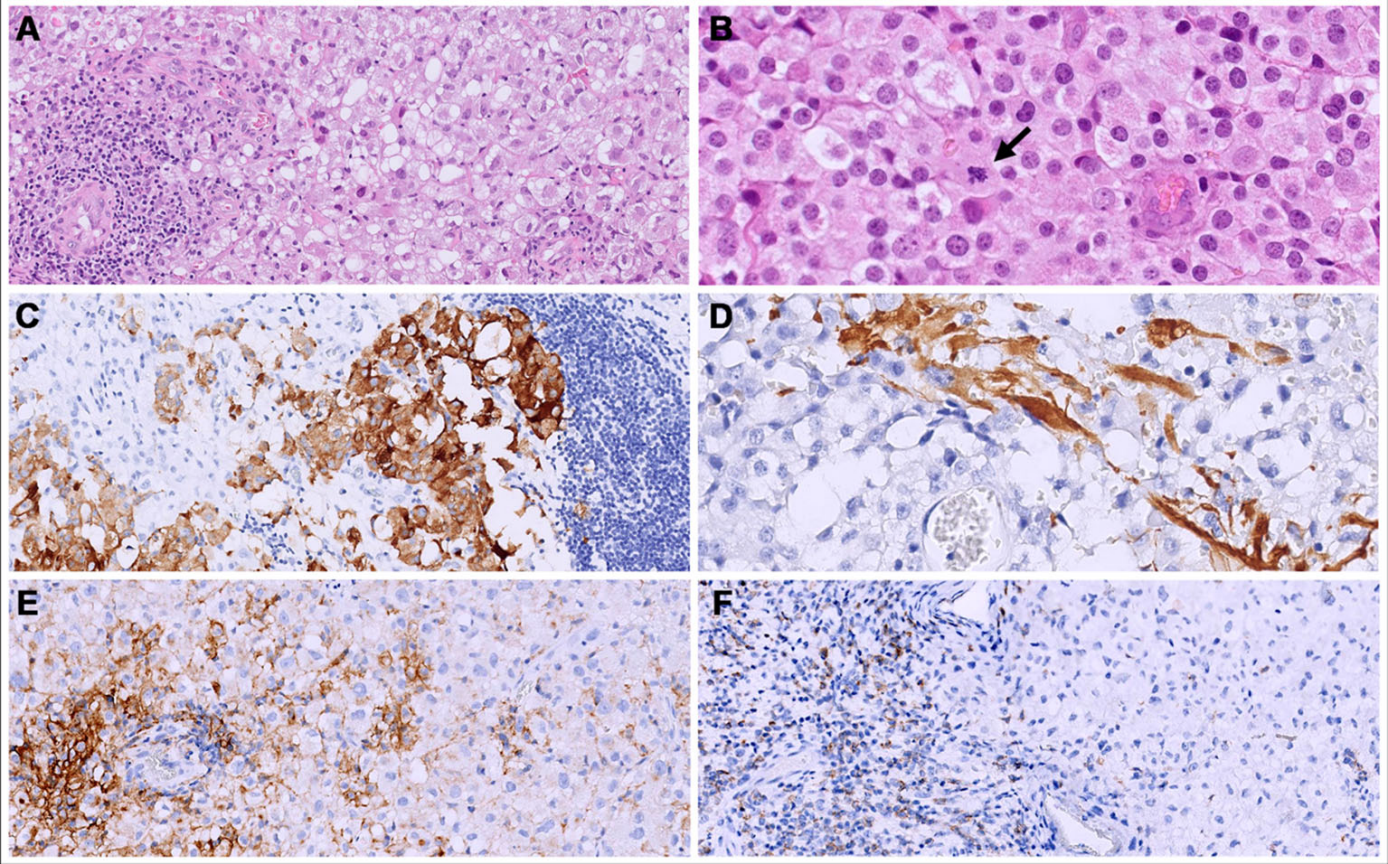
Both primary (cerebral) and metastatic (bone and lung) tumors consisted of solid sheets of epithelioid cells with clear to eosinophilic cytoplasm. **(A, B)** show the metastatic site, iliac bone); an abundant lymphocytic infiltrate was present mainly at the periphery of the tumors. **(B)** Mitoses were brisk (arrow). **(C, D)** The primary tumor (cerebral) showed a focal expression of synaptophysin **(C)** and GFAP **(D)**. **(E)** in the bone metastatic neoplasm, a membranous staining for PD-L1 was seen in 30% of the neoplastic cells. **(F)** PD-1 was expressed in 40% of the lymphocytes.

**Figure 6:**
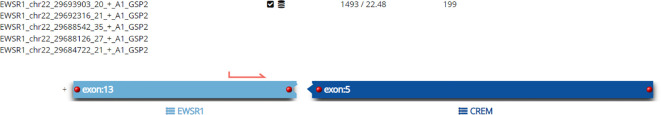
Analysis of the Archer™ FusionPlex Custom Panel-anchored multiplex PCR result showing an *EWSR1* exon 13 and *CREM* exon 5 gene fusion with reads (#/%) of 1493/22.48.

A 27-year-old woman was admitted to an outside hospital’s emergency department due to a headache and vomiting. She had no previous medical history or family history of cancer. Computed tomography (CT) and magnetic resonance imaging (MRI) revealed a mass on the right cerebellum that involved the transverse venous sinus (**Figure 1**). She underwent a partial resection, and the histological diagnosis highlighted a medulloblastoma. Following the diagnosis, the patient was referred to our center. The revised histological findings suggested the possibility of a high-grade glioneuronal tumor (HGG), which was later confirmed after a total secondary resection. Proton beam therapy (PBT) was administered at the surgical site with a total planned dose of 54 Grays (Gy) in 25 sessions, along with concomitant (75 mg/mq/day during PBT) and adjuvant (200 mg/mq/day for five days per month for six months) oral temozolomide. Subsequent MRIs showed complete remission of the disease. After 18 months from the suspension of treatment, a cerebral MRI revealed a local relapse at the surgical site (**Figure 2**). During the diagnostic workup, two metastatic lung lesions (the larger in the upper lingula segment) were discovered with suspicion of a lesion in the right iliac bone (**Figures 3**, **4**). The iliac bone biopsy confirmed the diagnosis of HGG, with 40% PD-1 expression on lymphocytes and 30% PD-L1 expression on neoplastic cells (**Figure 5**). Subsequently, immunotherapy with intravenous nivolumab (3 mg/kg/day) was started every two weeks and continued for two years without any reported toxicity. The patient achieved complete remission in all sites (**Figure 4D**), which was confirmed by a biopsy of the lesion in the right iliac bone, which showed only inflammatory tissue without evidence of neoplastic infiltration. After six months of discontinuing therapy, CT and a positron emission tomography (PET) scan revealed a relapse in the lung lesion and in the right iliac bone lesion (**Figures 3A**, **4**). A biopsy of this bone mass and a review of the previous cerebellar lesion allowed for a reevaluation of the entire case. Morphological features were similar in the primary and metastatic lesions: the tumor consisted of sheets of epithelioid cells with abundant eosinophilic to clear cytoplasm, large nuclei, and prominent nucleoli and showed a marked lymphoplasmacytic infiltrate at the periphery (**Figures 5A, B**). Mitoses were brisk (three mitoses/mm^2^), and the Ki67 proliferation index was approximately 15%. Although myxoid stroma and blood-filled cystic spaces were not prominent features, the expression of CD99 (membranous), GLUT-1 with a prominent paranuclear/Golgi pattern, focal EMA, and focal GFAP and synaptophysin raised a suspicion of an unusual mesenchymal tumor (**Figure 5**). A next-generation sequencing (NGS) panel (Archer Custom Fusion Plex Kit, Integrated DNA Technologies, IA) identified the presence of the *EWSR1::CREM* fusion transcript on both the primary tumor and the metastasis, confirming the diagnosis of metastatic intracranial mesenchymal tumor *FET::CREB* fusion-positive. (**Figure 6**).

In accordance with these findings, fluorescent *in situ* hybridization (FISH) demonstrated the rearrangement of *EWSR1*.

Additionally, a blood test for cancer predisposition syndromes was conducted using the Twist Custom Panel, which includes assessments for *CTNNB1, SMO, PIK3CA, PTEN, ID1, FGFR1, ARID1A, SMARCA4, CHD7, KDM4C, MYC, MYCN, MSH2, TP53, SUFU, PTCH1, PTCH2, ARID1B*, and *ERBB2* alterations, and no pathogenic/likely pathogenic variants were identified. Based on the previous clinical response to nivolumab, we decided to start a rechallenge treatment. After two cycles, nivolumab was prematurely discontinued due to the development of grade 2 immuno-mediated pneumonia, leading to a decrease in the patient’s performance status (ECOG 2) and iliac bone and lung disease progression. Consequently, PBT was performed on the right iliac bone lesion (with a total planned dose of 59.4 Gy in 48 sessions) with a complete surgical removal of the lung lesion. Thereafter, the patient received consolidation therapy with eight cycles of temozolomide plus irinotecan, achieving a complete remission of the disease.

**Figure 7:**
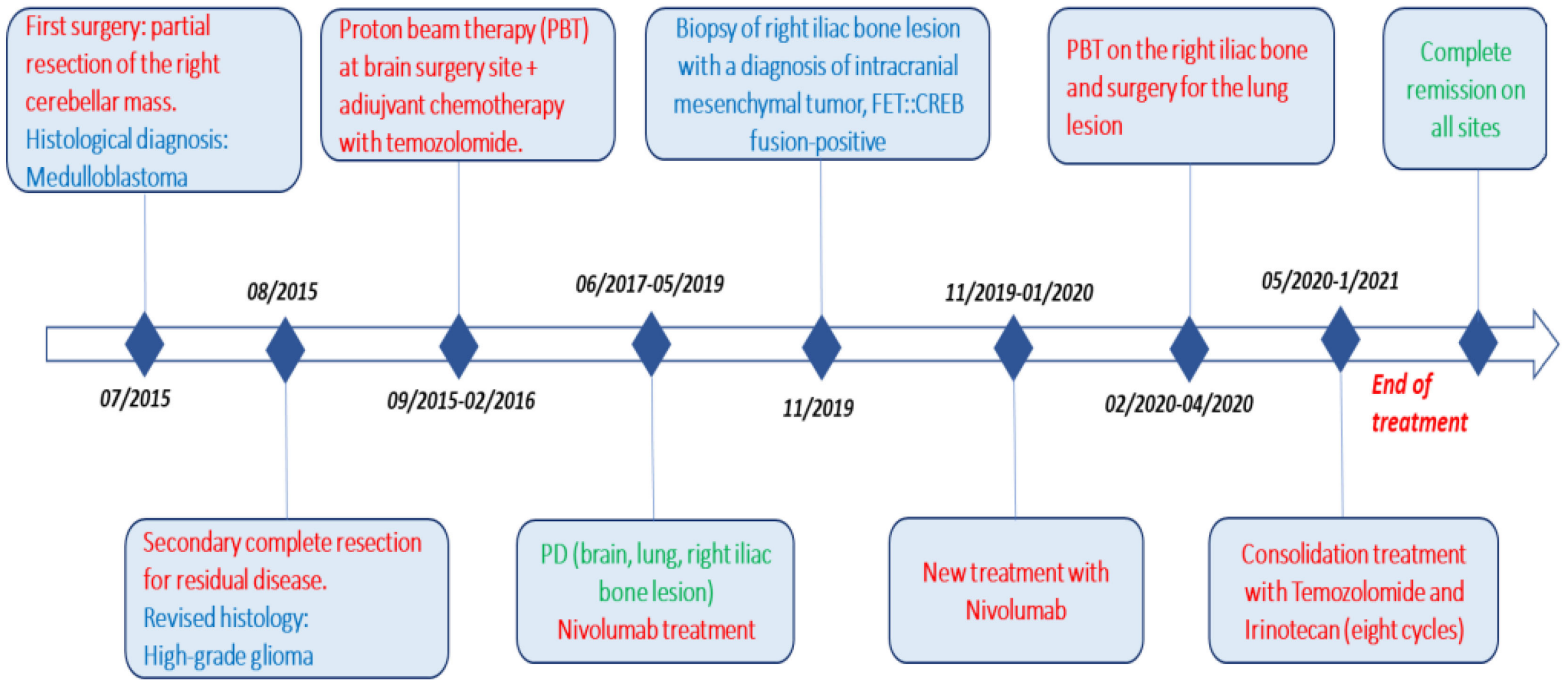
Complete timeline, including pathways to reach the diagnosis (surgery) and treatment (red), different histologies (blue), and response to therapy (green).

She is currently still in complete remission with an optimal quality of life, nine years after diagnosis and two years after discontinuation of therapy (**Figure 7**).

## 3 Discussion

AFH is a rare mesenchymal tumor (0.3% of all soft tissue tumors) (3), defined by a fusion of a *FET* family gene (usually *EWSR1*, but rarely *FUS*) with members of the cAMP Response Element-Binding Protein family (ATF1, CREB1, or CREM) (9). These specific gene fusions play a key role in AFH tumorigenesis (9–11). In 1979, Enzinger described “angiomatoid malignant fibrous histiocytoma” as a new histological entity for the first time (12, 13). Although AFH has been known for more than 40 years, only in 2021 was it included in the WHO CNS classification as an intracranial mesenchymal tumor (13). In fact, AFH usually occurs in the extremities of young adults, more frequently in the second decade of life, with a female prevalence, and is rarely diagnosed in the CNS (14, 15). Clinically, patients with AFH can experience, in addition to local symptoms, systemic non-specific signs such as fever, anemia, and weight loss (6, 7, 16). Regional recurrence rates after surgery are relatively low (approximately 15%), but AFH can occasionally metastasize (the most common sites are lymph nodes, lungs, and liver), especially if it is not completely removed (5, 16).

CNS-AFH represents a rare primary site (**Tables 1**, **2**), and the longest patient outcomes reported in the literature are five years with a median progression-free survival of 28 months (8, 9, 11). Patients with subtotal resection showed a local recurrence within 12 months. At microscopic histology examination, AFH is characterized by multinodular proliferation of spindle-shaped, or round cells with syncytial growth, forming bundles surrounded by fibrous pseudocapsules, pericapsular lymphoplasmacytic cuffing, and pseudovascular spaces full of blood. Most AFH cases express desmin but lack positivity for myogenin or MyoD, EMA, and CD68 (4, 29). Regrettably, the definitive histological diagnosis of AFH still presents a challenge (3). In the literature, several unusual clinicopathological presentations and histological variants are described (7, 9). The intracranial mesenchymal tumor is one of these variants, characterized by a prominent collagenous stroma with a dense intracellular matrix resembling the myofibroblastic tumor, poorly differentiated carcinoma, or meningioma. Not all cases contain a myxoid matrix (1, 3, 10).

**Table 1:** Published cases of conventional AFH (NA, not available; GTR, gross total resection; STR, subtotal resection; CHT, chemotherapy; XRT, radiotherapy; CP angle, cerebellopontine angle).

Authors	Age/Gender	Location	Surgery	Genetic molecular	After surgery treatment	Time to relapse (m)	Post relapse treatment	Follow-up (m)
Dunham et al., 2008 (17)	25/M	Occipital lobe	GTR	*EWSR1::ATF1*	None	NA	NA	NA
Ochalski et al., 2010 (18)	35/M	Temporal lobe	GTR	negative	None	0.8	Two radiological ablations	49
Hansen et al., 2015 (19)	17/M	Parieto-occipital lobe	GTR	negative	None	NA	NA	3
Alshareef et al., 2016 (20)	58/F	Porous trigeminus	GTR	negative	NA	NA	NA	6
Konstantinidis et al., 2019 (21)	13/F	Frontal lobe	GTR	*EWSR1::ATF1*	None	60	Surgery	132
	12/F	Frontal lobe	STR	*EWSR1::CREM*	None	28	NA	28
Sloan et al., 2021 (9)	12/M	Frontal lobe	STR	*EWSR1::ATF1*	XRT (59.4Gy)	6; 10 (local recurrence)	Surgery	24
	9/F	Frontal lobe	STR	*EWSR1::ATF1*	None	3	Surgery	63
	24/F	Occipital lobe	NA	*EWSR1::ATF1*	NA	NA	NA	NA
	13/F	Frontal lobe	GTR	*EWSR1::ATF1*	None	None	None	24
	34/F	Tentorium	STR	*EWSR1::ATF1*	None	54	Surgery	81
	17/F	CP angle	STR	*EWSR1::ATF1*	XRT (59.4 Gy); CHT	12 (metastasis to thoracic lymph nodes and vertebrae)	NA	27
	70/M	CP angle and Spinal cord	STR	*EWSR1::ATF1*	None	Continuous progression	None	1
	17/F	CP angle	STR	*EWSR1::ATF1*	None	None	None	13
	14/F	Lateral ventricle	GTR	*EWSR1::CREB1*	None	None	None	59
	39/F	Lateral ventricle	GTR	*EWSR1::CREB1*	None	None	None	6
	10/M	Frontal lobe	GTR	*EWSR1::CREB1*	None	9 (local recurrence)	None	57
	14/F	Lateral ventricle	GTR	*EWSR1::CREB1*	None	None	None	47
	25/F	CP angle	GTR	*EWSR1::CREB1*	None	11 (local recurrence)	None	30
	14/F	Parieto‐occipital lobe		*EWSR1::CREB1*	None	49 (local recurrence)	None	57
	12/M	Tentorium	GTR	*EWSR1::CREB1*	None	None	None	46
	15/F	Spinal cord	STR	*EWSR1::CREM*	XRT (dose unknown)	6 (local recurrence)	None	30
	14/F	Lateral ventricle	GTR	*EWSR1::CREM*	None	None	None	38
	5/F	Frontal lobe	GTR	*EWSR1::CREM*	CHT	2;6 (local recurrence x 2)	Surgery	11
	3/M	Frontal lobe	STR	*EWSR1::CREM*	XRT (54 Gy)	None	None	6
	4/F	Occipital lobe	GTR	*FUS::CREM*	None	9; 13 (local recurrence x 2)	None	36
Vizcaino et al., 2020	50/F	Bifrontal falcine	GTR	*EWSR1::ATF1*	None	NA	NA	NA
Ward et al., 2020 (15)	48/F	Lateral ventricle	GTR	*EWSR1::CREB1*	None	4	Surgery and XRT	12
Aguiar, 2020 (14)	58/F	Lateral ventricle	GTR	*EWSR1::CREB1*	None	None	None	6
Lopez-Nunez et al., 2020	20/F	Posterior fossa	GTR	*EWSR1::CREM*	None	None	None	54

**Table 2:** Published cases of myxoid mesenchymal AFH (NA, not available; GTR, gross total resection; STR, subtotal resection; CHT, chemotherapy; XRT, radiotherapy; CP angle, cerebellopontine angle).

Authors	Age/gender	Location	Surgery	Genetic molecular	After surgery treatment	Time to relapse (m)	Post relapse treatment	Total # of follow-ups (m)
Kao et al., 2017 (10)	15/F	Meninges	NA	*EWSR1::CREM*	NA	None	None	17
	23/F	Meninges	NA	*EWSR1::CREM*	None	NA	NA	NA
	20/M	Frontal lobe	NA	*EWSR1::CREM*	NA	NA	NA	NA
	12/M	Frontal lobe	NA	*EWSR1::CREM*	NA	NA	NA	NA
	23/F	Meninges	NA	*EWSR1::CREM*	None	NA	NA	NA
	20/M	Frontal lobe	NA	*EWSR1::CREM*	NA	NA	NA	NA
	12/M	Frontal lobe	NA	*EWSR1::CREM*	NA	NA	NA	NA
Bale et al., 2018 (3)	12/M	Posterior cerebellum fossa	STR	*EWSR1::CREM*	None	NA	None	12
	14/F	Intraventricular	STR	*EWSR1::CREM*	None	NA	None	12
	18/M	Frontal lobe	STR	*EWSR1::CREM*	None	NA	None	12
Sciot et al., 2018 (22)	17/M	Frontal lobe	GTR	*EWSR1::ATF1*	None	3	Second surgery and XRT	84
Gareton et al., 2018 (23)	19/M	Tentorium cerebelli	GTR	*EWSR1::CREM*	None	120	NA	120
Spatz et al., 2018 (24)	22/F	Occipital lobe	STR	na	NA	None	NA	3
Ghanbari et al., 2019 (6)	58/F	Parafalcine	STR	*EWSR1::CREM*	None	NA	NA	3
Gunness et al., 2019 (25)	32/F	Temporal lobe	STR	na	None	12	Surgery	24
White et al., 2019 (26)	9/M	Frontal lobe	GTR	*EWSR1::CREM*	None	6	Surgery, XRT	6
Ballester et al., 2020 (27)	67/M	Temporal lobe	STR	*EWSR1::ATF1*	None	None	None	3,5
Komatsu et al., 2020 (28)	53/F	Third ventricle	STR	*EWSR1::CREB1*	Gamma knife surgery	None	None	3
Lopez-Nunez et al., 2020	17/M	Parietal lobe	GTR	*EWSR1::CREB1*	None	None	None	2

The intracranial mesenchymal tumor’s radiological aspect shows hypointense T1 signal and hyperintense T2 signal lesions with strong enhancement after gadolinium administration (10). Differential diagnoses include meningioma and schwannoma (**Table 3**) because intracranial mesenchymal tumors mimic an extra-axial lesion with homogeneous contrast enhancement and a small dural tail in T1 fluid-attenuated inversion recovery (FLAIR) (3). Furthermore, the expression of glial and neural markers is a pitfall for glial and glioneuronal tumors.

**Table 3:** Differential diagnosis.

Microscopical examination	Most common genomic features	Histological features	Immunohistochemical features
Intracranial mesenchymal *FET::CREB* fusion-positive tumor	• *EWSR1::CREB1* • *EWSR1::CREM* • *FUS::CREM* • No *TERT* promoter mutations or other modifications in genes known to be altered in meningiomas (*NF2, TRAF7, KLF4, AKT1, SMO, PIK3CA, SMARCB1, BAP1, YAP1*). *(* 9, 30)	• Collagenous stroma with dense intercellular matrix; multinodules of epithelioid/rhabdoid cells; stellate/spindle-shaped cells with syncytial growth, forming bundles surrounded by fibrous pseudocapsules and pericapsular lymphoplasmacytic cuffing. • Pseudovascular spaces filled with hemangioma-like blood. Cellular whorls resembling those of meningioma are present. Mitotic activity is generally low, typically less than five mitoses per 1 mm^2,^ and necrosis is not a common feature (9).	• Desmin expression, EMA, GLUT-1, and CD99 are expressed in a membranous pattern. S100, synaptophysin, CD68, and MyoD immunostaining are present in a subset of intracranial mesenchymal tumors. • Negativity for cytokeratins AE1/AE3 and CAM5, expression of the glial fibrillary acidic protein (GFAP), myogenin, somatostatin receptor 2A (SSTR2A), and HMB45. • Abundant intercellular basement membrane deposition. • The Ki-67 labeling index is low (less than 5%, occasionally elevated up to 15- 20%). There is no association of specific CREB fusion partners with distinct Ki-67 labeling indexes (9, 10).
Meningioma	• *NF2* mutation, *PIK3CA, TRAF7/AKT1*, and *SMO* in meningothelial or transitional meningioma • *KLF4/TRAF7* mutations in the secretory meningioma component, *SMARCE1* in the clear cell subtype, • *BAP1* in rhabdoid and papillary subtypes. • *TERT* promoter mutation and/or homozygous deletion of *CDKN2A/B*, H3K27me3 loss of nuclear expression (31, 32).	Spindle-shaped and round or oval nuclei with finely dispersed chromatin. The cytoplasm of the cells is eosinophilic and may contain small eosinophilic granules called psammoma bodies (31).	Positive for EMA, SSTR2A,PR, vimentin, and S100 protein. EMA, S100, and collagen IV are present in fibrous meningiomas, and GLUT-1 in angiomatous meningiomas. Ki-67 is a prognostic marker and a predictor of tumor growth rate and prognosis (31).
Schwannoma	*NF2, SMARCB1 LZTR1, LAST1, LAST2* (31, 33).	Spindle-shaped cells are arranged in patterns called Antoni A and Antoni B. Antoni A areas consist of compact, highly cellular areas with spindle-shaped cells arranged in a palisade pattern, while Antoni B areas consist of loosely arranged spindle-shaped cells in a myxoid stroma with fewer cell nuclei (33, 34).	• Positive for S100 protein and are enveloped by a pericellular basal lamina, containing laminin and collagen type 4 (130). • Positive for SOX10 and (GFAP); immunoreactive tumors also express cytokeratins (33, 34). • Ki-67 is always low and is associated with tumor growth rate or recurrence.

Tauziède-Espariat et al. described 11 cases of CNS mesenchymal tumors with *FET::CREB* fusion. Six in total were a specific cluster with DNA methylation, and five showed no relation to any of the other classes but were similar to the clusters of extra-CNS angiomatoid fibrous histiocytomas, clear cell sarcomas, or solitary fibrous tumors. Therefore, the authors demonstrated that intracranial *FET::CREB*-fused tumors did not present a single molecular tumor entity but a primary intracranial mesenchymal tumor, the *FET::CREB*-fused family (4). Several other authors reported small groups of intracranial mesenchymal cases: Kao et al. described four children and young adults diagnosed with intracranial mesenchymal tumors with myxoid component and *EWSR1::CREB1*, *EWSR1::CREM*, or *EWSR1::ATF1* fusions (10); Bale et al. described three pediatric cases with similar histology and fusions (3); Sloan et al. reported a series of 20 cases of intracranial mesenchymal tumors with *FET::CREB* fusion and comprehensively characterized their radiologic, molecular, and clinicopathologic features. Several other intracranial mesenchymal tumor cases without myxoid component and *EWSR1::CREB1* fusion have been reported in the literature (**Tables 1**, **2**).

The reported treatment was surgical in all cases; nevertheless, adjuvant radiotherapy and sarcoma-based regimens have also been reported (2). Our case confirms that local treatment (including surgery and proton beam therapy) shows the most favorable outcomes and a more promising prognosis (35).

In the case of the patient described, it was initially difficult to make the correct diagnosis, but by expanding the immunohistochemical panel and integrating it with molecular data, the correct diagnosis of intracranial mesenchymal tumor *FET::CREB* fusion-positive was made. Due to the rarity of the histopathological and molecular features, this case was previously published as part of a series of *EWSR1*-rearranged intracranial tumors (29). At the onset, we treated our patient according to the diagnosis of HGG. However, the uncommon occurrence of extracranial metastasis led us to identify a personalized treatment approach. We conducted molecular analysis, which revealed high levels of PD-1 and PD-L1 expression. This information allowed us to initiate treatment with nivolumab, making our case the first to be documented to receive immunotherapy in the medical literature. As a result of this therapy, the patient achieved and maintained a partial response for nearly three years. However, it was only after the most recent disease progression and five years after the initial diagnosis that we were able to identify an intracranial mesenchymal tumor with the *EWSR1::CREB1*fusion transcript. After this diagnosis, the patient received local treatments (surgery for the lung lesion and proton therapy for the bone lesion) along with consolidation with systemic drug therapy. This consolidation regimen consisted of temozolomide and irinotecan, tailored to sarcoma-specific protocols, with good tolerability and outpatient administration.

In conclusion, our case highlights the necessity and mandatory molecular studies workup for these rare diseases, which are crucial for refining personalized therapies and exploring novel treatment options.

## Data availability statement

The original contributions presented in the study are included in the article/supplementary material. Further inquiries can be directed to the corresponding author.

## Ethics statement

Written informed consent was obtained from the individual(s) and/or minor(s)’ legal guardian/next of kin for the publication of any potentially identifiable images or data included in this article.

## Author contributions

AM: responsible for all published work, FD: responsible for writing paper write, SR, IG, RA, ACan, and MG: critical revision of histological and molecular case diagnosis. ACac and GM: examination, diagnosis and follow-up of the patient, and critical revision of the manuscript. ACar and GMM: critical revision of the manuscript for intellectual content. SC: radiological diagnosis and follow-up of the patient. SV: treatment and follow-up of the patient, and critical revision of the manuscript. All authors contributed to the article and approved the submitted version.
